# Interactions Between Kidney Function and Cerebrovascular Disease: Vessel Pathology That Fires Together Wires Together

**DOI:** 10.3389/fneur.2021.785273

**Published:** 2021-11-24

**Authors:** Sandro Marini, Marios K. Georgakis, Christopher D. Anderson

**Affiliations:** ^1^Department of Neurology, Boston Medical Center, Boston University School of Medicine, Boston, MA, United States; ^2^Institute for Stroke and Dementia Research, University Hospital of LMU Munich, Munich, Germany; ^3^McCance Center for Brain Health, Massachusetts General Hospital, Boston, MA, United States; ^4^Program in Medical and Population Genetics, Broad Institute, Cambridge, MA, United States; ^5^Department of Neurology, Brigham and Women's Hospital, Boston, MA, United States

**Keywords:** stroke, genetic epidemiology, chronic kidney disease, intracerebral hemorrhage, small vessel disease (SVD)

## Abstract

The kidney and the brain, as high-flow end organs relying on autoregulatory mechanisms, have unique anatomic and physiological hemodynamic properties. Similarly, the two organs share a common pattern of microvascular dysfunction as a result of aging and exposure to vascular risk factors (e.g., hypertension, diabetes and smoking) and therefore progress in parallel into a systemic condition known as small vessel disease (SVD). Many epidemiological studies have shown that even mild renal dysfunction is robustly associated with acute and chronic forms of cerebrovascular disease. Beyond ischemic SVD, kidney impairment increases the risk of acute cerebrovascular events related to different underlying pathologies, notably large artery stroke and intracerebral hemorrhage. Other chronic cerebral manifestations of SVD are variably associated with kidney disease. Observational data have suggested the hypothesis that kidney function influences cerebrovascular disease independently and adjunctively to the effect of known vascular risk factors, which affect both renal and cerebral microvasculature. In addition to confirming this independent association, recent large-scale human genetic studies have contributed to disentangling potentially causal associations from shared genetic predisposition and resolving the uncertainty around the direction of causality between kidney and cerebrovascular disease. Accelerated atherosclerosis, impaired cerebral autoregulation, remodeling of the cerebral vasculature, chronic inflammation and endothelial dysfunction can be proposed to explain the additive mechanisms through which renal dysfunction leads to cerebral SVD and other cerebrovascular events. Genetic epidemiology also can help identify new pathological pathways which wire kidney dysfunction and cerebral vascular pathology together. The need for identifying additional pathological mechanisms underlying kidney and cerebrovascular disease is attested to by the limited effect of current therapeutic options in preventing cerebrovascular disease in patients with kidney impairment.

## Introduction

Chronic kidney disease (CKD) affects around 10% of the general population globally and has an increasing prevalence, posing a major burden on public health systems (1). While the effects of kidney dysfunction on cardiovascular disease have long been explored, recent literature has provided evidence for the role of kidney disease in both early and advanced stages of cerebrovascular atherosclerosis and cerebral small vessel disease (SVD) (2). However, the mechanisms underlying associations between CKD and cerebrovascular disease have been underinvestigated (3). In this narrative review, we highlight work exploring the intersection of chronic kidney disease and cerebrovascular disease. We also discuss pathophysiological features connecting vascular damage in the two organs and summarize epidemiological data supporting the effect of CKD on acute and chronic manifestations of cerebrovascular disease. Furthermore, we provide an overview of recent genetic findings that support these associations, suggest possible new pathological pathways combining kidney and brain disease, and summarize data that may help to disentangle correlations from causal associations between the two organs. Finally, we discuss therapeutic options for patients suffering from chronic kidney disease, among whom cerebrovascular disease treatment is under recognized and insufficiently treated.

## Hemodynamic Properties of the Kidney and Brain Vasculature

Both the kidney and the brain are high-flow end organs that receive blood through the renal arteries and the carotid and vertebrobasilar circulation, respectively. Their microvasculature is composed of small arteries, penetrating arterioles, capillaries, and venules, overall referred to as small vessels. The small vessels of the kidney and the brain are unique as their cells receive continuous high-volume flow throughout systole and diastole against very low vascular resistance.

Given the particular anatomy and physiology of small vessel circulation, kidney and brain tissue are susceptible to the same microvascular insults in response to aging and exposure to vascular risk factors. As a result, these two tissues are more prone to developing what is commonly referred as SVD (4). This term encompasses a range of pathological processes including fibrosis, development of inclusions in the basement membrane, and hyalinization of the vessel wall with the final common effect of narrowing of the vascular lumen leading to stenosis or occlusion and ultimately ischemia (5). Hypertension and hyperglycemia are the two most common vascular risk factors leading to SVD (6). Early studies showed that increasing blood pressure (BP) and consequently pulsatile stress led to tearing of endothelial and smooth muscle cells within small arteries, causing disruption to the vessel (7). Similarly, hyperglycemia, especially in tissue with specific high flow needs, leads to changes in insulin signaling, oxidative stress, and inflammation which promote the progression of microvascular pathologies (8).

It has been hypothesized that the pathophysiology of SVD in the kidney is similar to that of the brain. The same features of small arterial dilations and aneurysms as well as lipohyalinosis and fibrinoid necrosis are seen in the brain as in the kidneys. Because of these shared hemodynamic properties between the brain and the kidney, kidney disease has been proposed to progress in parallel with cerebrovascular pathology and particularly SVD.

## Epidemiology of Kidney Dysfunction and Cerebrovascular Disease: Associations With Stroke

Several epidemiological observations support the hypothesis that kidney impairment is associated with higher risk of stroke, independent of the etiological subtype. When assessing the association between kidney dysfunction and stroke, studies have used reduced estimated glomerular filtration rate (eGFR), albuminuria, and CKD diagnosis as indices of kidney dysfunction. GFR, representing the process of ultrafiltration of plasma from glomerular capillaries into Bowman's space, is estimated from serum concentrations of endogenous filtration markers, such as creatinine or cystatin via equations which account for non-GFR related factors (9). The glomerular capillary wall generally blocks the passage of albumin and other large serum proteins. An increase in the normal albumin excretion rate is called albuminuria and reflects an alteration in structure of the glomerular capillary wall (9). Levels of albumin ranging from 30 to 300 mg in a 24-h urine collection are referred to as microalbuminuria and represent a relatively early marker of kidney disease. Macroalbuminuria is defined as a urinary albumin excretion of ≥300 mg/24 h. CKD is defined by having more than 3 months of decreased eGFR or evidence of kidney damage including albuminuria (9).

In the Northern Manhattan Study, a decreased eGFR (defined throughout this section as <60 ml/min/1.73 m^2^) was associated with a 2.5-fold higher risk of developing stroke [hazard ratio (HR) = 2.65; 95% confidence interval (CI) = 1.47–4.77] (10). Similar associations were observed in the prospective Atherosclerosis Risk in Communities study, where subjects with decreased eGFR had nearly double the risk of stroke (HR = 1.81; 95% CI = 1.26–2.02), even after adjustment for conventional vascular risk factors (11). A meta-analysis which included 284,672 individuals also found a higher relative risk [RR] for developing incident stroke among those with decreased eGFR (RR = 1.43; 95% CI = 1.31–1.57) (12). Similarly, in the largest meta-analysis conducted to date with over 5 million individuals, participants with decreased eGFR had an increased stroke risk (RR = 1.73; 95% CI = 1.57–1.90) (13). Interestingly, the association was attenuated after adjustment for multiple BP measurements, but still remained significant (RR = 1.10; 95% CI = 1.02–1.18). Lastly, the European Rotterdam Study followed 5,993 community-dwelling individuals for 11.6 years and found a 10% increased incidence of any stroke (HR = 1.11; 95% CI = 1.01–1.23) per standard deviation (SD) decrease in creatinine-based eGFR. Similar results were obtained when eGFR was assessed *via* cystatin-C.

As for other indices of kidney disease, albuminuria has been independently associated with risk of stroke in a dose-dependent manner. A study with almost 50,000 individuals found the presence of microalbuminuria to almost double the risk of stroke independently of other cardiovascular risk factors (14). In a meta-analysis, both micro- and macroalbuminuria were associated with higher risk of incident stroke (RR = 1.58 and RR = 2.65, respectively) (15). Interestingly, recovering from microalbuminuria is associated with a slight reduction but not normalization of risk of cardiovascular events [individuals with regression from microalbuminuria had a HR of 2.62 (95% CI = 1.95–3.54)] (16).

However, the major pathophysiological and causal differences between stroke etiologies highlight the need for a more elaborate exploration of the effects of kidney disease on stroke risk across the different stroke subtypes. Such analyses could provide deeper insight into the underlying mechanisms and define the right patient subgroups for developing preventive strategies. Stroke is divided into ischemic stroke and intracerebral hemorrhage (ICH). The main sources of ischemic stroke include large artery atherosclerosis, cardioembolism, and cerebral SVD. Although a recent epidemiologic study designed to assess whether CKD is associated with a specific stroke subtype failed to find that CKD increases risk of any individual etiology (17), associations between CKD and different mechanisms of ischemic stroke can be inferred by several studies.

The strength of association between kidney function and cerebrovascular disease highlights a pressing need to determine appropriate timing of screening assessments of kidney function. In the United States alone, 14%-15% of individuals aged 20 years or older suffer from some form of kidney dysfunction (18). Patients with vascular risk factors have an even higher risk of developing renal dysfunction, with downstream implications for cerebrovascular events. A comprehensive determination of vascular risk should therefore include assessment of renal function.

### Cardioembolic Stroke

CKD appears to increase the risk of cardioembolic stroke predominantly through atrial fibrillation (AF). Population-based studies have confirmed a higher prevalence of AF among patients with CKD. For example, the prevalence of AF was up to 3 times higher in The Chronic Renal Insufficiency Cohort than in the general population (19). CKD triggers several mechanisms which may result in an increased risk for AF. Renin-angiotensin-aldosterone dysfunction, chronic inflammation, vascular calcification, and left ventricular hypertrophy all increase the risk of cardioembolic stroke through AF as its predisposing risk factor (20). Beyond AF, CKD also increases the risk of thromboembolic events. A meta-analysis reviewing 25 studies of patients with AF and end stage renal disease (ESRD) showed that the presence of severe kidney impairment doubles the risk of stroke (21). Another study showed that kidney dysfunction (defined as either reduced eGFR or proteinuria) is associated with a higher incidence of thromboembolism independent of other stroke risk factors (HR = 1.39; 95% CI = 1.31–1.71 for eGFR <45 and HR = 1.54; 95% CI = 1.29–1.85 for proteinuria) (22). Taken together, this evidence suggests that CKD increases the risk for developing AF as well as the risk of thromboembolic events in the context of AF, both of which increase the risk for cardioembolic stroke.

### Large Artery Stroke

CKD is an established risk factor for atherosclerosis, and as such may increase the risk of large artery stroke (LAS) via promoting extra- and intracranial atherosclerosis. In the Japanese Suita Study of urban residents, CKD was associated with carotid artery stenosis [adjusted odds ratio (OR) = 3.16; 95% CI = 2.05–4.88] independent of hypertension (23). Similarly, in the Intervention Project on Cerebrovascular Diseases and Dementia, a community-based cohort study with 3,364 participants, individuals in the lower quartile of eGFR had the greatest increase in carotid intima-media thickness (2.4%; 95% CI = 2.0–2.7%), an ultrasound marker of carotid atherosclerosis (24).

Arterial stiffness represents another clinical measure proven to be independently predictive of fatal and non-fatal cardiovascular events and as such is a useful surrogate end point for cardiovascular disease outcomes (25). It reflects the functional and structural changes in the vascular wall and correlates with atherosclerosis of the large arteries often involved in LAS (26). Even mildly impaired renal function is associated with increased arterial stiffness and subsequent independent increase in stroke risk (27, 28). This association between renal impairment and large artery atherosclerosis results in an increased risk of atherosclerotic ischemic stroke.

In the CHOICE study, which evaluated stroke in dialysis patients, the overall stroke incidence was almost ten times the incidence in the general population, with large-vessel atherosclerosis found in 11% (29). In a Japanese study of 639 subjects, severe kidney dysfunction almost doubled the risk of the atherosclerotic stroke (OR = 1.81; 95% CI = 1.23-2.68) (30). A recent study which followed subjects after a transient ischemic attack (TIA) or minor stroke found that microalbuminuria was associated with recurrent events and significant internal carotid artery stenosis (OR = 3.4; 95% CI = 2.2–5.2) independent of other vascular risks factors (31).

### Small Vessel Stroke

There are few epidemiological studies which have specifically assessed the contribution of CKD to the SVD subtype of ischemic stroke, commonly referred to as lacunar infarction. The Cardiovascular Health Study reported a linear association between decreasing kidney function and prevalence of lacunar infarction (OR = 1.20; 95% CI = 1.09–1.32 for each SD of decreased cystatin C clearance) after multivariable adjustments (32). Similar ORs were also reported in the Rotterdam Scan Study for decreased eGFR (33). These findings were confirmed in a meta-analysis which showed a nearly three-fold increased risk of silent cerebral infarctions in patients with low eGFR (OR = 1.77; 95% CI = 1.36–2.11) (34). In contrast, in a retrospective observational study of 639 patients with stroke and ESRD, severe kidney dysfunction was associated with atherothrombotic stroke (OR = 1.81; 95% CI = 1.23–2.68) and cardioembolic stroke (OR = 2.25; 95% CI = 1.32–3.83) and showed an OR of similar magnitude for lacunar stroke, which however did not reach the level of statistical significance (OR = 1.67; 95% CI = 0.98–2.84) (30). While some studies may not have found an excess risk of lacunar stroke in CKD patients, associations have been reported for SVD neuroimaging features beyond lacunar stroke. The United Kingdom Young Lacunar Stroke DNA Study recruited 1,023 patients with lacunar infarction. In this study, decreased eGFR did not represent a risk factor for multiple lacunar infarcts vs. isolated lacunar infarcts, but increased the risk of moderate/severe white matter hyperintensity (WMH), a marker of chronic cerebral SVD (35). Similarly, a meta-analysis including 37 publications and 20,379 subjects calculated the risk of having renal impairment for patients with lacunar stroke compared to patients with non-lacunar stroke. Although no specific association between renal impairment and lacunar stroke was found (OR = 0.88; 95% CI = 0.6–1.30), the presence of SVD features on imaging was associated with worse renal function (36).

### Embolic Stroke of Undetermined Source (ESUS)

Available data of kidney function in patients with ESUS are limited and few studies assess the relationship between CKD and ESUS. In the largest ESUS dataset, patients with intact or impaired renal function had the same risk of suffering from ESUS (37). As such, whether kidney dysfunction could lead to ESUS or other forms of cryptogenic stroke remains uncertain.

### Intracerebral Hemorrhage

CKD increases the risk of ICH. Up to 46% of patients presenting with ICH are also affected by CKD (38, 39). In a retrospective cohort study of 516,197 adults, individuals with CKD had almost double the RR of hemorrhage (including ICH) (RR = 1.9; 95% CI = 1.5–2.4) when compared to subjects with normal kidney function (21). A prospective population-based cohort study in China found that subjects with proteinuria had almost double the risk of ICH (HR = 1.90; 95% CI = 1.35–2.67) compared to those without. This association was confirmed after adjustment for established cardiovascular risk factors for proteinuria, but not for low eGFR (40). A cohort study conducted with more than 10,000 people with ESRD found a similar increase in risk of ICH when compared to the general population (41). Concordant findings were also reported in a population-based study from UK primary care (42). In contrast, a more recent retrospective cohort study in South Korea which analyzed almost 200,000 subjects did not find any association between eGFR and ICH (43).

The high comorbidity between ICH and CKD is in part attributable to the burden of hypertension. Around 30% of patients affected by CKD have elevated ambulatory BP with normal office BP (masked hypertension), and up to 40% suffer from resistant hypertension (44). Therefore, the impact on risk of ICH is likely substantial considering that patients with a higher than normal systolic BP had a 5.5 (95 % CI = 3.0–10.0) fold increased rate of ICH compared with normotensive individuals (45).

Other hypothesized mechanisms include metabolic derangements common in advanced renal disease, such as high serum phosphate and low serum calcium levels. Calcium and phosphorus are pivotal in the maintenance of cell function in the endothelium and vascular smooth muscle, hence their abnormality can result in endothelial dysfunction and impairment of cerebral autoregulation (46). In a prospective study of patients on dialysis, when comparing patients with different levels of phosphate, every 1 mmol/L increase in serum phosphate corresponded to a two-fold increased risk of developing ICH (HR = 2.07; 95% CI = 1.10–3.81) (47). Lastly, patients suffering from CKD may exhibit uremia, which affects the balance between platelet adhesion activators and inhibitors, resulting in a net activation defect. Uremia also reduces platelet receptor glycoprotein, leading to impaired platelet adhesion to the sub-endothelium (48). As a consequence, patients with advanced CKD are at increased risk of hemorrhage (49, 50).

Further, CKD can increase the risk of ICH via the use of AF-related anticoagulants. As discussed earlier, the incidence and prevalence of AF in patients with CKD are higher than in the general population. A systematic meta-analysis showed that AF in patients with ESRD increased the risk of stroke, ICH and mortality, although with a high degree of variability (21).

## Epidemiology of Kidney Dysfunction and Cerebrovascular Disease: Associations With Cerebral Small Vessel Disease

Beyond acute manifestations of cerebrovascular disease, there are multiple studies supporting associations between kidney dysfunction and neuroimaging markers of subclinical chronic cerebral SVD. Classical neuroimaging biomarkers for SVD, such as WMH, silent lacunes and cerebral microbleeds each have evidence for association with SVD and are themselves worthy of further exposition outside the context of the present review. Briefly, a meta-analysis of 31 studies and 23,056 participants found microalbuminuria to be associated with a higher risk of all markers of cerebral SVD including WMH (OR = 1.70; 95% CI = 1.43–2.01), lacunes (OR = 1.86; 95% CI = 1.49–2.31), cerebral microbleeds (OR = 1.78; 95% CI = 1.30–2.43), and enlarged perivascular spaces (OR = 1.78; 95% CI = 1.02–3.09 in the basal ganglia, and OR = 3.27; 95% CI = 1.49-7.20 in the centrum semiovale) (51). Similarly, several other studies have found linear associations between eGFR and markers of cerebral SVD in the general population (12). Nonetheless, on the basis of these conventional study designs, it is not possible to delineate whether the reported associations represent causal effects of kidney dysfunction on the development or progression of cerebral SVD or if they are the result of a shared pathology, such as systemic endothelial and microvascular dysfunction that underlies both kidney and cerebral SVD.

## Mechanisms Through Which Renal Dysfunction Leads to Cerebral Small Vessel Disease and Other Cerebrovascular Events

**Figure 1:**
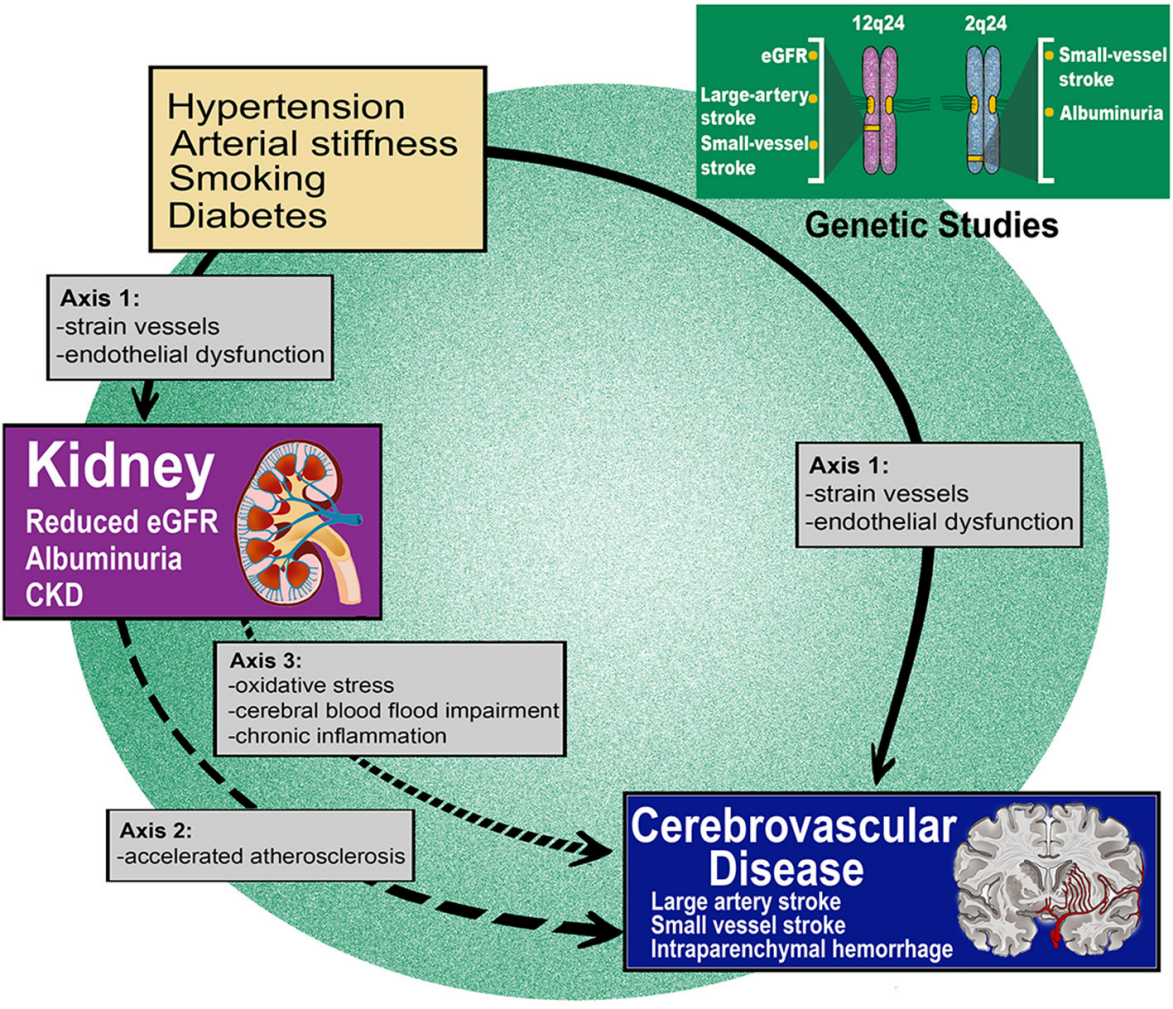
Renal dysfunction is robustly associated with acute and chronic forms of cerebrovascular disease. Mechanisms through which renal dysfunction leads to cerebral small vessel disease and other cerebrovascular events can be summarized in three axes. Genetic epidemiology findings reinforce the causality of the highlighted connections.

The complex relationship between kidney dysfunction and cerebrovascular disease can be summarized in three axes: (axis 1) shared unique susceptibility to vascular risk factors and the same resultant tissue pathology; (axis 2) over-representation and accentuation of vascular risk factors in patients with renal disease; (axis 3) sequelae of renal disease playing a role in stroke pathogenesis (Figure 1). These axes are theoretical and serve to illustrate complex relationships and pathways with several points of contact between them.

### Strain Vessel Hypothesis (Axis 1)

The “strain vessel hypothesis,” based on hypertensive vascular damage, has been suggested as a possible mechanism explaining the association between CKD and stroke (52). Kidney and brain share commonalities in terms of vascularization, as previously discussed. Both organs have vessels therefore referred to as “strain vessels,” as they are systems with low vascular resistance allowing continuous high-volume perfusion (53). These features make the kidney and the brain extremely vulnerable to hypertension. In fact, high BP not only impacts the histopathology of arterioles leading to a replacement of smooth muscle cells by lipohyalinosis, but also impacts the hemodynamics of large arteries mostly via arterial stiffness which in turn exacerbates the deleterious effects on strain vessels (54). As a result, these cerebral subcortical perforating arteries and renal juxtamedullary afferent arterioles lose their autoregulation and thus impair regional blood flow. Furthermore, damage to renal juxtamedullary afferent arterioles leads to glomerular hypertension and sclerosis as well as impaired downstream circulation in the vasa recta and medulla (55). The first causes a progressive loss of renal function, while the second affects the sodium balance with an overall summative effect on worsening systemic hypertension (55).

### Endothelial Dysfunction (Axis 1)

Albuminuria occurs when podocyte density decreases below a certain threshold. It represents a marker of glomerular barrier impairment, specifically glomerular endothelium damage. Given the similarities in the anatomy of the kidney and brain, it has been hypothesized that the endothelial dysfunction which occurs at the glomerular level and causes albuminuria happens simultaneously at a systemic scale (56, 57). In this sense the presence of albuminuria reflects a more generalized impairment of vascular endothelial function, and the vascular leakage which occurs as a consequence will ultimately impair tissue perfusion with resultant ischemia. In the Framingham Heart Study, any level of urinary albumin excretion was associated with progressively increased cardiovascular risk and mortality, independent of diabetes or hypertension (58).

Additional evidence is provided by research on derivatives of the aminoacid L-arginine, such as asymmetric dimethylarginine (ADMA) and symmetric dimethylarginine (SDMA). ADMA and SDMA are elevated in patients with kidney dysfunction as well as in patients with other SVD manifestations, independent of classical vascular risk factors (59). ADMA levels were found to correlate with the extent of WMH, risk of small vessel disease, and were found to be elevated in subjects affected by heritable forms of SVD such as Cerebral Autosomal Dominant Arteriopathy with Sub-cortical Infarcts and Leukoencephalopathy (CADASIL) (59). L-arginine derivatives, similar to albuminuria, may represent biomarkers of the same systemic endothelial pathology shared by the kidney and the brain.

### Accelerated Atherosclerosis (Axis 2)

Accelerated atherosclerosis has been described in patients with kidney dysfunction. Dyslipidemia is common in patients affected by CKD. As a result of proteinuria and lipoprotein transport impairment, CKD patients often have an increase in serum triglycerides, very-low-density lipoprotein, and low-density lipoprotein (LDL) cholesterol, all of which have significant atherogenic potential (60, 61). Hypercholesterolemia may also be worsened by the upregulation of 3-hydroxy-3-methylglutaryl CoA reductase in patients with CKD (62).

Modified lipoproteins from elevated LDL have been hypothesized to trigger innate immune reactions, which result in inflammation and accelerated diastasis of lipids in the intima of the vessel. Additionally, CKD has been described as a pro-inflammatory state, and enhanced levels of fibrinogen and matrix metalloproteinases have been variably described in this patient population. Inflammation is known to affect atherosclerotic lesions' stability and risk of rupture.

Instability and rupture of atherosclerotic carotid plaques have been found to be significantly higher in patients with CKD compared to patients with normal renal function in a retrospective study of patients undergoing carotid endarterectomy (83 vs. 52%, *p* = 0.001, and 59 vs. 36%, *p* = 0.039, respectively) (63). However, data showing that patients with CKD have higher percentages of calcification and lower collagenous content of carotid plaques when compared to subjects with normal renal function represent an important counterfactual observation, as heavily calcified plaque is typically considered less prone to rupture and cause ischemia (64).

Taking this evidence together, increased atherosclerosis in CKD patients is likely to arise in the setting of combined insults of dyslipidemia and accentuated inflammation (65, 66). Concomitantly, higher levels of factor VIII and von Willebrand factor, which have been described in CKD patients as compared to the normal population, may further increase the risk of thrombotic and atheroembolic events.

Closely related to atherosclerotic changes, even moderate stages of CKD have been associated with changes in the biomaterial and biological characteristics of the vessels, resulting in arterial stiffness (67). Most likely via impaired renal excretion of vascular toxins and metabolic derangements of the calcium homeostasis, kidney disease promotes vascular calcification and eventually arterial stiffness. A prospective study evaluating patients with mild to moderate CKD found that progression to ESRD was an independent determinant of carotid stiffness (HR = 2.48; 95% CI = 1.63–3.78) (68), with the above mentioned impact on stroke risk and cardiovascular events.

### Cerebral Blood Flow Impairment (Axis 3)

Cerebral vasculature is able to maintain stable cerebral blood flow (CBF) despite changes in BP. Impaired kidney function can lead to impaired cerebral autoregulation and subsequently make brain perfusion more strictly dependent on systemic BP (69, 70). In this scenario, patients with CKD are at risk for both cerebral hypoperfusion and hyperperfusion depending on systemic pressure. This disconnection between tissue perfusion and metabolic demand can be measured by deviation from the expected CBF. Demonstrating this autoregulatory failure, studies have reported that patients with CKD have both higher and lower CBF compared to patients with normal kidney function. In the Systolic Blood Pressure Intervention Trial, reduced kidney function was independently associated with higher global and white matter CBF (71). In the Rotterdam Study, lower eGFR was independently associated with lower CBF (0.42 ml/ min/100 ml decrease in CBF for each standard deviation of eGFR reduction) (72).

Impairment of CBF may be a repercussion of an effect of kidney disease on nitric oxide, which is crucial in vascular responsivity. As discussed above, kidney impairment increases ADMA and SDMA, which are inhibitors of nitric oxide synthase, and therefore have been implicated in vascular disease (73). Although limited by small sample size, studies have shown that doses of ADMA increase vascular stiffness and decrease cerebral perfusion in healthy subjects, and that increased concentrations of ADMA are associated with LAS and cardioembolic stroke (59, 74).

Lastly, CKD can also lead to CBF dysregulation and hence stroke through anemia. Anemia in fact is a well-known complicating feature of CKD, present in up to two thirds of subjects with severe kidney impairment (75). Concomitantly, anemia has been shown to increase the risk of stroke by almost 50%, possibly through impairment of oxygen delivery to tissue, blood supply and CBF (76). This relationship between anemia and CBF may explain the further increase in stroke risk observed in CKD patients with anemia, compared to CKD patients without anemia (11).

### Oxidative Stress (Axis 3)

Chronic low-grade inflammation can be seen in patients at all stages of CKD. Levels of pro-inflammatory molecules Interleukin (IL)-6, IL-1 and Tumor Necrosis Factor (TNF)-α are higher even in patients in the early stages of CKD, and this inflammation appears to influence CKD progression. As CKD worsens, uremic toxins, indoxyl sulfate, and guanidino compounds enter the central nervous system and promote neuroinflammation *via* macrophage and microglia polarization toward a pro-inflammatory phenotype (77).

Patients with CKD suffer from impaired production of many antioxidant sources, such as glutathione peroxidases and mitochondrial superoxide dismutase (78), with commensurate increases in uremic toxins such as indoxyl sulfate, p-cresyl sulfate, advanced glycation end products, oxidized LDL, and activated Nicotinamide Adenine Dinucleotide Phosphateoxidase (79). In this context, it is perhaps unsurprising that levels of biomarkers for oxidative stress (such as malondialdehyde) have been found to be increased in patients suffering from CKD and cardiovascular disease (80). Finally, iron therapy, frequently required in advanced stages of kidney dysfunction as a result of chronic disease anemia, may also contribute to oxidative stress (81).

This oxidative stress and consequent inflammation have profound vascular effects and influence on cerebral blood flow. Free radicals increase endothelial permeability, platelet aggregation, and alter reactivity to vasodilators (82). Oxidative stress eventually contributes to endothelial dysfunction, arterial stiffness and development of atherogenesis as discussed above in patients with CKD (83).

### Impairment of Blood–Brain Barrier Function (Axis 3)

The blood–brain barrier (BBB) is made of vascular endothelium, a specialized basement membrane, astrocyte foot processes, and pericytes. Due to this specialized barrier, access to the brain from the blood is highly regulated. Animal experiments have demonstrated impairment of BBB integrity in the setting of both acute kidney injury and CKD (84). This BBB disruption can lead to leakage of toxic compounds and proteins into perivascular tissues. The consequent edema, arteriolar stiffening and impaired vasoregulation can contribute to hypo-oxygenation, hypoperfusion, and ultimately ischemic changes. Each of these features has been identified in SVD and chronic cerebrovascular diseases (85, 86).

### The Role of Human Genetic Studies in Clarifying the Effects of Kidney Dysfunction on Cerebrovascular Disease

The aforementioned observational studies and many others have recognized the role of CKD in cerebrovascular disease and quantified the associations between the two. However, conventional analytical methods in observational research are limited in providing evidence for causal effects, and therefore insights about the mechanisms underlying the observed associations are constrained. In particular, it remains unclear whether both the kidney and the brain suffer concomitantly from similar injuries and ultimately perfusion failure or whether damage to one can lead to injury in the other. Human genetics can provide valuable information about the causal networks underlying CKD and cerebrovascular pathologies. Genetic variation is determined at conception and is therefore not influenced by confounding processes occurring later in life. Thus, studying genetic data can provide useful additional and orthogonal anchors to causality.

Conventional genetic research, genome wide association studies (GWAS) and post-GWAS research instruments, such as Mendelian randomization (MR), may clarify the complex relationship between the two diseases. In parallel with the axes inferred by epidemiological studies, genetic data may be used to test the following hypotheses: kidney dysfunction and cerebrovascular disease are the result of a single shared biological defect, as is the case in rare monogenic diseases (axis 1); kidney dysfunction and cerebrovascular disease share common pathologic pathways that contribute to both diseases (axis 2); kidney disease causally contributes to the risk of specific cerebrovascular pathologies that increase the risk of stroke (axis 3).

#### Axis 1

The presence of similar underlying susceptibility between kidney disease and stroke is supported by rare diseases in which a genetic mutation results in clinical presentation of concurrent kidney impairment and stroke. One example is Anderson-Fabry Disease, which is characterized by accumulation of glycosphingolipids. Deposits of glycosphingolipids in the vascular endothelia and smooth muscle cells cause vessel stenosis or occlusion. Stroke may result from either direct vessel involvement or cardioembolism. As the disease progresses, renal vasculature becomes affected as well, and failure occurs almost inevitably (87). Collagen IV (*COL4A1*) mutations have been described in clinical syndromes with invariable involvement of the eye, kidney, and brain. *COL4A1* mutations result in vessel abnormality and SVD with stroke and CKD (87). Lastly, patients affected by CADASIL, caused by a mutation on the *Notch3* gene, may display renal injury together with high risk of ischemic stroke (87). These clinical observations and genetic studies support the presence of an underlying organic pathway, the dysfunction of which leads to physiologic disarray in both the renal and cerebrovascular systems.

The hypothesis of accentuated vascular damage in patients with renal disease (axis 1) is further upheld by findings from genetic epidemiology. Recent GWAS have identified variants in genes and associated pathways which may contribute to alteration of renal function (88, 89). Among these, the *APOL1* gene has been consistently linked to severe hypertension-induced kidney impairment in African American individuals (90). Although the underlying mechanisms remain unclear, in a setting of hypertensive kidney injury, *APOL1* downregulation alters podocyte function and hastens glomerulosclerosis with secondary deleterious effects on BP and vascular risk (91).

#### Axis 2

Large-scale human genetic studies have contributed to the study of the relationship between kidney and cerebrovascular disease (92). Polygenic risk scores (PRS), which combine genetic variants associated with a specific disease or trait, have proven to be a valuable tool to determine an individual's susceptibility to that disease across a normalized continuum of risk. In prior work, PRS capturing genetic predisposition for lower eGFR have been associated with increased risk of stroke related to large artery atherosclerosis. Similarly, PRS reflecting predisposition to microalbuminuria have been associated with increased risk of stroke related to large artery atherosclerosis and SVD (93). A more recent study corroborated the genetic correlation between renal dysfunction and cerebrovascular disease and confirmed the shared heritability and genetic predisposition between CKD and risk of ischemic stroke (94). This study further dissected the shared pathogenesis among stroke subtypes, identifying that it is predominantly the genetic predisposition toward lower eGFR that drives associations with higher risk of LAS and SVD stroke (94). These genetic findings support the hypothesis that there are shared pathogenic mechanisms between the two diseases.

By applying a pairwise GWAS analysis, which explores shared genomic signals between two traits at the single locus level (95), genomic loci which may be involved in the shared pathogenesis of two diseases can be highlighted (94). Using this approach, this same study identified a locus at chromosome 12q24 which was found to be associated with both eGFR and LAS risk (94). Two genes in this region may highlight the mechanisms underlying this shared pathogenesis: *ATXN2*, which is involved in spinocerebellar ataxia type 2 and is associated with kidney disease, and *SH2B3*, which is associated with hypertension and vascular disease. These results reinforce the hypothesis that, once in place, the same pathologic pathways can drive both renal and cerebrovascular manifestations. The same study also demonstrated that the observed shared genetic pathways act independently of genetic susceptibility to hypertension. Taken together, these data compliment prior work in supporting the hypothesis that CKD, as estimated by impaired eGFR is associated with LAS risk and lend orthogonal support to the epidemiology theory that a different axis (axis 2) links the kidney and the brain beyond the already known shared susceptibility to vascular risk factors.

Similar inferences can be advanced regarding SVD. In the aforementioned genetic study, common genetic variants at 2q33 were associated with risk of small vessel stroke, CKD and severity of WMH. Three genes (*NBEAL1, FAM117B* and *WDR12*) within the 2q33 locus were found to be expressed in various cells of the nervous system including astrocytes, oligodendrocytes and neurons (96). Variants in the *WDR12* gene have also previously been associated with WMH burden, further corroborating these results (97, 98). Taken together, these data support the possibility that there are specific biological pathways, potentially involving the products of these genes, which once perturbed may lead to SVD pathology in those organs where small vessels are particularly represented, namely kidney and deep brain structures.

#### Axis 3

To explore whether the identified associations could also represent causal effects of kidney disease on the cerebral vasculature, bioinformatics methods have been applied to derive causal inference from genetic associations in a form of instrumental variable analysis (94). MR uses genetic variants associated with a trait as instruments and explores their effects on the outcome of interest (99). By anchoring on genetic variants, which are randomly allocated at conception and thus not influenced by confounders, MR can help elucidate causal relationships, as has been specifically demonstrated in stroke over the last several years (100). MR analysis has shown that genetic predisposition to CKD conveys a mildly increased risk of all types of stroke (OR = 1.07; 95% CI = 1.01–1.15). Assessing continuous indices of kidney dysfunction and subtypes of stroke, genetically determined lower eGFR and genetically determined microalbuminuria were found to increase risk of LAS by almost two-fold. Finally, genetically elevated microalbuminuria increased the risk of ICH, although the wide confidence interval of this finding makes the true estimate of excess risk difficult to ascertain (OR = 5.09; 95% CI = 1.02–26.41). In reverse, none of the cerebrovascular traits were found to have a causal link to any of the studied measures of kidney impairment. These MR findings support the presence of potentially causal mechanisms (possibly ones identified as axis 3), which when triggered by kidney dysfunction will increase the risk of cerebrovascular disease. To further corroborate this hypothesis, many of the genes found to be associated with risk of CKD (88, 89) are involved in biological mechanisms already identified by epidemiological and experimental observations; increased oxidative stress in the renal tubules, impaired function of podocytes, and altered renal hemodynamics are mechanisms also supported by recent GWAS findings (101).

## Therapeutic Options

There are no specific treatments aimed at reducing stroke risk in patients with CKD (102). Given the pathogenic processes described above, optimization of established vascular risk factors, which trigger the cascade leading to deterioration of the microvascular structure of the kidney and brain is currently the most principled strategy available.

Reducing abnormally high pulsatile stress in cerebral and renal small vessels may lead to an improvement in the risk of stroke. Studies on the effects of calcium channel blockers and angiotensin converting-enzyme inhibitors, which reduce arterial stiffness and hence pulsatile stress, have shown evidence of superiority to conventional diuretics and β-blockers in progression of microvascular disease (103–105).

The China Stroke Primary Prevention Trial evaluated the effect of BP modifiers in hypertensive patients with mild-to-moderate CKD. After multivariable adjustment and independent of medication adherence, systolic BP variability increased the risk of first stroke (HR = 1.41; 95% CI = 1.17–1.69) in this category of patients (106). Excessive lowering of BP can also be detrimental. The Secondary Prevention of Small Subcortical Strokes trial with 2,454 participants showed rapid kidney function decline in the lower-BP-target arm compared to the higher-target arm (OR = 1.40; 95% CI = 1.07–1.84). These data suggest the need for careful long-term BP monitoring in CKD patients and establishment of more refined BP targets beyond standard monotonic manometric values (107).

In terms of antiplatelet or anticoagulant therapies, there are no specific recommendations to reduce stroke risk in patients with CKD. However, patients with CKD have both high thromboembolic risk and high bleeding risk. Patients with CKD may also have altered responsiveness to antiplatelet drugs and enhanced bleeding complications with antithrombotic treatment (108, 109). As such, weighing the balance of risks and benefits of antithrombotic or anticoagulant treatment is particularly challenging in this category of patients. The benefit of stroke prevention from warfarin use has conflicting evidence, and the adoption of novel oral anticoagulants in populations with advanced renal impairment is operationally challenging due to altered pharmacokinetics in the presence of reduced creatinine clearance (110). Similarly, limited data are available for stroke prevention with dual antiplatelet therapy in CKD patients. Studies have demonstrated the safety of dual antiplatelet therapy after coronary stenting in CKD patients, with no difference in 1-year composite outcomes (including all-cause death and major bleeding) when compared to patients with normal renal function (111). More specifically for cerebrovascular disease, two recent trials which showed benefit in stroke recurrence with short term dual antiplatelet therapy after minor stroke or TIA, did not exclude patients based on their kidney function (112, 113).

Statins have not been shown to reduce stroke risk in advanced CKD patients. A meta-analysis that included data from the largest randomized controlled trials showed that statins did not impact stroke risk in dialysis patients (114). Similarly, the Pravastatin Pooling Project, which included 4,491 patients with severe kidney disease, concluded that there was no statistically significant difference between statin and placebo groups in the prevention of stroke (102, 115).

Sodium-glucose cotransporter-2 inhibitors were initially developed to reduce hyperglycemia in diabetic patients, and seem to have beneficial cardiometabolic effects in patients with CKD, independent of diabetes (116). In the EMPA-REG OUTCOME trial, empagliflozin reduced non-fatal stroke risk by 14% when compared against placebo (HR = 0.86; 95% CI = 0.74–0.99), together with other primary cardiovascular endpoints (117). These vascular benefits in patients with declining kidney function appear to be largely independent from the effects on glycemia or hypertension, and warrant further investigation as preventive agents.

Lastly, a novel approach involves the use of anti-inflammatory therapies to reduce cardiovascular risk. Given the systemic pro-inflammatory status of CKD patients, efforts have specifically tested the benefits of anti-inflammatory compounds in this high-risk group. The CANTOS trial showed a reduction in vascular endpoints among patients with coronary artery disease receiving canakinumab, a monoclonal antibody against IL-1b (118). Furthermore, the RESCUE trial showed a reduction of inflammation and thrombosis biomarkers in subjects with CKD receiving a monoclonal antibody against IL-6, compared to placebo (119). Given evidence from human genetic and observational studies implicating IL-6 signaling in ischemic stroke risk (120, 121), anti-inflammatory approaches may represent a useful target for reducing stroke risk in CKD patients in the near future.

Regarding acute therapies, both intravenous thrombolysis with recombinant tissue plasminogen activator (IV-rtPA) and intra-arterial thrombectomy have been associated with worse overall outcome in patients with acute stroke and advanced CKD compared to patients with normal renal function (122). Increased bleeding risk and reduced efficacy of thrombolysis in patients with CKD have been suggested as mechanisms of unfavorable outcomes (123–125). Additional caution should be applied as these data are derived from studies of small sample size, and prediction of outcomes is complex in CKD patients as they tend to suffer from a higher burden of co-comorbidities. A recent systematic review of more than 53,000 patients clarified that impaired renal function independently associates with mortality after IV-rtPA administration, but has not been associated with a higher rate of hemorrhagic conversion (126). Similarly for endovascular procedures, limited data are available regarding safety in patients with CKD. Two studies showed that the presence of severe kidney impairment was a predictor of poorer functional recovery and higher mortality, but not through increased rates of hemorrhagic complications (127, 128). Given the paucity of data on safety and patient outcomes in CKD patients, the presence of CKD alone should not constitute a reason to defer IV-rtPA administration or endovascular thrombectomy.

Despite the lack of specific primary prevention of cerebrovascular disease in patients with CKD, the strength of the association between kidney disease and stroke supports development of rational strategies for noninvasive screening of kidney function, to fully capture individual risk for cerebrovascular events.

## Future Directions

Given the prevalence of kidney dysfunction and its association with stroke, the impact on healthcare is massive and underlines the need for screening interventions and novel therapies. Elucidating mechanisms of shared pathobiology and risk between the kidney and cerebrovascular system is pivotal to developing new strategies to ameliorate both stroke and progressive kidney disease in patients at risk for these conditions. Genetic epidemiology in particular represents a useful means to help clarify the temporality and interactions among the hypothesized pathological mechanisms, with pleiotropy analyses demonstrating genes linking kidney and cerebrovascular damage, and MR analyses offering clues about the directionality of associations between kidney and brain pathology (129). Building on the works of ongoing population-based research and cohort studies, the construction of larger and more powerful genetic association studies may identify new mechanisms underlying the biological pathways of renal and cerebral SVD, leading to novel targets for disease prevention for patients with these prevalent and highly morbid conditions (130, 131).

## Author Contributions

SM and MKG: literature search and manuscript writing. CA: manuscript writing and review. All authors contributed to the article and approved the submitted version.

## Funding

MKG was supported by a Walter-Benjamin fellowship from the German Research Foundation [Deutsche Forschungsgemeinschaft (DFG), GZ: GE 3461/1-1] and the FöFoLe program of LMU Munich (FöFoLe-Forschungsprojekt Reg.-Nr. 1120). CA was supported by the NIH (R01NS103924 and U01NS069763), the American Heart Association (812095, 18SFRN34150007), and Massachusetts General Hospital.

## Conflict of Interest

CA receives sponsored research support from Bayer AG, and has served as a consultant for ApoPharma. The remaining authors declare that the research was conducted in the absence of any commercial or financial relationships that could be construed as a potential conflict of interest.

## Publisher's Note

All claims expressed in this article are solely those of the authors and do not necessarily represent those of their affiliated organizations, or those of the publisher, the editors and the reviewers. Any product that may be evaluated in this article, or claim that may be made by its manufacturer, is not guaranteed or endorsed by the publisher.
